# Therapeutic management of resectable mismatch-repair deficient/microsatellite instability-high gastroesophageal adenocarcinoma: a real-world analysis from Austria

**DOI:** 10.1007/s10120-026-01776-1

**Published:** 2026-07-23

**Authors:** Martin Korpan, Daniel Nothdurfter, Bernhard Doleschal, Lukas Weiss, Sophie Roider-Schur, Leopold Öhler, Ewald Wöll, Julia M. Berger, Elisabeth S. Bergen, Gerald W. Prager, Behrang Mozayani, Anna S. Berghoff, Matthias Preusser, Hannah C. Puhr, Florian Huemer, Aysegül Ilhan-Mutlu

**Affiliations:** 1 Division of Oncology, Department of Medicine I, Medical University of Vienna, Vienna, Austria; 2 Christian Doppler Laboratory for Personalized Immunotherapy, Department of Medicine I, Medical University of Vienna, Waehringer Guertel 18-20, 1090 Vienna, Austria; 3 Department of Internal Medicine I for Hematology With Stem Cell Transplantation, Hemostaseology, and Medical Oncology, Ordensklinikum Linz, Linz, Austria; 4 Department of Internal Medicine III With Hematology, Medical Oncology, Hemostaseology, Infectiology and Rheumatology, Oncologic Center, Paracelsus Medical University, Salzburg, Austria; 5 Department of Medicine I, Oncology, St. Josef Krankenhaus, Vienna, Austria; 6 Department of Internal Medicine, St. Vinzenz Hospital Zams, Zams, Tyrol Austria; 7 Department of Pathology, Medical University of Vienna, Vienna, Austria

**Keywords:** Gastroesophageal adenocarcinoma, Mismatch repair deficiency, Microsatellite instability-high, Immune checkpoint inhibition, Non-operative management

## Abstract

**Background:**

Mismatch-repair deficient (dMMR)/microsatellite instability-high (MSI-H) gastroesophageal adenocarcinoma (GEA) is associated with favorable prognosis but limited benefit from perioperative chemotherapy. Although immune checkpoint inhibition (ICI)-based approaches have shown promising activity in early-phase studies, the optimal therapeutic combinations and the feasibility of non-operative management (NOM) in resectable disease remain unclear.

**Methods:**

This exploratory, retrospective, multicenter, real-world study included 55 patients with resectable dMMR/MSI-H GEA, who were divided into subgroups, characterized and compared according to treatment strategy (chemotherapy, chemoimmunotherapy, immunotherapy and initial resection). Pathological and clinical complete response (pCR, cCR), progression-free (PFS) and overall survival (OS) were evaluated.

**Results:**

Patients received chemotherapy (n=10), chemoimmunotherapy (n=16), immunotherapy (n=13), or initial resection without subsequent systemic treatment (n=16). Forty five (82%) patients had surgical resection. The pCR rate was 22% (n=2/9) in the chemotherapy, 27% (n=4/15) in the chemoimmunotherapy, and 100% (n=5/5) in the immunotherapy (i.e., doublet ICI) subgroup, respectively. The overall cCR rate in NOM patients was 33% (n=3/9), with rates of 37.5% (n=3/8) in the immunotherapy subgroup and 0% (n=0/1) in the chemoimmunotherapy subgroup. Complete response rates (pCR/cCR) were significantly associated with PD-L1 CPS ≥10 (p=0.027).

**Conclusion:**

This real-world study is, to our knowledge, the first to evaluate a broad spectrum of therapeutic combinations in patients with resectable MSI-H/dMMR GEA, showing meaningful antitumor activity, particularly with doublet ICI. Given the challenges of conducting randomized prospective trials in this setting, such real world data may support clinicians in guiding therapeutic decision-making.

**Supplementary Information:**

The online version contains supplementary material available at https://doi.org/10.1007/s10120-026-01776-1.

## Background

Gastroesophageal tumors are a major contributor to global disease burden with the majority already presenting in an advanced stage [1, 2]*.* In resectable gastroesophageal adenocarcinoma (GEA), patients benefit from multimodal treatment strategies, with surgery and perioperative chemo(immuno-)therapy constituting the standard of care in curative-intent therapy [3–5].

Mismatch-repair deficient (dMMR)/microsatellite instability-high (MSI-H) GEA is associated with better outcome than mismatch repair proficient/microsatellite stable tumors [6]. Since the approval of anti-programmed cell death protein 1 (PD-1) and its ligand (PD-L1) inhibitors across various tumor entities, dMMR/MSI-H has emerged as the most robust predictive factor of immune checkpoint inhibition (ICI) in solid tumors [7, 8]. As the dMMR/MSI-H phenotype only occurs in around 4–22% of GEA, predominantly in operable disease and in older patients with potentially more comorbidities, this subpopulation remains underrepresented in randomized controlled trials [2, 9–12].

To date, ideal management of resectable dMMR/MSI-H GEA remains unclear, as this subgroup was not prospectively analyzed in the trials leading to the approval of perioperative chemotherapy, and some available subgroup analyses suggest no benefit compared with surgery alone [6, 13].

Early-phase studies investigating neoadjuvant or definitive immunotherapy, including dual immune checkpoint blockade, have reported high pathological complete response (pCR) rates in selected patients with resectable dMMR/MSI-H GEA [14–16]. In case of clinical complete response (cCR), a definitive immunotherapy-based approach was investigated to pursue a non-operative management (NOM) in cohort 2 of the INFINITY trial [15]. Nonetheless, response rates vary widely between studies, underscoring the influence of treatment strategy, patient selection, and limited sample size [9]. Given the rarity of this subgroup, early-phase clinical trials are frequently designed as single arm studies, which precludes comparison between potential different treatment strategies [9].

Consequently, insights from randomized controlled trials largely rely on exploratory analyses of dMMR/MSI-H subgroups reported in larger studies, such as KEYNOTE-585 and MATTERHORN [17, 18]. No survival benefit was observed in the KEYNOTE-585 trial, however, the MSI-H subgroup appeared to derive some benefit from the addition of immunotherapy [19]. In contrast, survival data from the MATTERHORN study regarding the MSI-H subgroup have not been published yet and are currently only available for the overall population [5].

In light of these evolving treatment possibilities and due to the inefficiency of adjuvant chemotherapy/immunotherapy [20, 21], the 2024 European Society for Medical Oncology (ESMO) living guidelines recommend omission of adjuvant therapy for patients with localized resectable dMMR/MSI-H GEA after initial resection, while neoadjuvant chemotherapy may be considered in selected cases to achieve tumor downstaging [22].

Recent real-world and retrospective data suggest promising activity of immunotherapy-based definitive and neoadjuvant approaches in localized dMMR/MSI-H GEA [23, 24]. However, comparative data regarding all possibly investigated treatment strategies remain limited.

Therefore, we designed this retrospective, multicenter study to gain insights into clinical characteristics and outcome of patients with resectable dMMR/MSI-H GEA based on the treatment strategy, i.e. perioperative chemotherapy, perioperative chemoimmunotherapy, perioperative immunotherapy, and initial resection with no prior or subsequent systemic treatment.

## Methods

### Study design

This exploratory, retrospective, multicenter, real-world study included patients treated at the following Austrian institutions between January 1, 2014 and August 15, 2025: *Division of Oncology, Department of Medicine I, Medical University of Vienna; Department of Internal Medicine III with Haematology, Medical Oncology, Haemostaseology, Infectiology and Rheumatology, Paracelsus Medical University, Salzburg, Austria; Department of Internal Medicine I for Hematology With Stem Cell Transplantation, Hemostaseology and Medical Oncology, Ordensklinikum Linz, Linz, Austria; Department of Internal Medicine, Krankenhaus St. Vinzenz Zams; 1st Department of Internal Medicine/Oncology at St. Josef Hospital.*

### Patient recruitment

Patients with localized, resectable dMMR/MSI-H GEA were eligible for inclusion. Eligible patients were aged ≥ 18 years, had no prior antitumor therapy, and presented with clinical stage I–III disease as per local tumorboard decretion. A histopathologically confirmed diagnosis of esophageal, gastroesophageal junction, or gastric adenocarcinoma was required and had to be established by expert pathologists. Tumors had to be classified as dMMR, defined by loss of expression of one or more mismatch repair proteins (MLH1, MSH2, MSH6, PMS2) on immunohistochemistry (IHC), or as MSI-H based on polymerase chain reaction (PCR) or next-generation sequencing (NGS). Mortality data were collected from hospital records and supplemented with public records from Statistik Austria to ensure completeness. Survival data were analyzed up to December 14, 2025. For patients without a documented date of death as of this cut-off, survival was censored at the date of the last known visit to the treating hospital.

### Patient classification

According to therapy strategies and recommendations at the time of treatment start, patients were divided into four main groups:

1. Patients with pre- or perioperative chemotherapy,
2. Patients with non-, pre- or perioperative chemoimmunotherapy,
3. Patients with non-, pre- or perioperative immunotherapy, and
4. Patients with initial resection (including minimally invasive procedures such as endoscopic mucosal resection or endoscopic submucosal dissection) and no prior or subsequent systemic treatment.

NOM was defined as the absence of resection of the primary tumor, irrespective of initial intention.

Treatment initiation and subsequent management decisions were based on interdisciplinary tumor board consensus at each participating center. Decisions took into account clinicopathological characteristics, performance status, patient preference, available scientific evidence and the respective standard of care at the time of initial cancer diagnosis. Subsequent management decisions, particularly regarding surgical resection, additionally considered tumor response to systemic therapy.

### Response rate assessment

Primary endpoints were pCR in resected patients and cCR in patients with NOM. pCR was defined as the absence of histopathological residual tumor specimen, assessed with the ypTNM classification or with the tumor regression grade by Mandard [25] or Becker [26]. As there is no guideline-based definition of cCR in the context of NOM in GEA and clinical experience remains limited, we defined cCR as the absence of radiologically detectable residual tumor on either positron emission tomography–computed tomography or contrast-enhanced computed tomography, the absence of endoscopically/endosonographically detectable residual tumor, and no histological evidence of residual tumor in re-biopsy specimens.

### Statistical analysis

For descriptive statistics, continuous variables were presented as median (interquartile range), and categorical variables were summarized using percentages. Median follow-up was estimated using the reverse Kaplan–Meier method. Associations between treatment regimens, complete response rates, and clinical or tumor characteristics were assessed using the chi-square test/Fisher’s exact test or the Wilcoxon/Kruskall-Wallis rank-sum test, as appropriate. OS was calculated from the date of initial cancer diagnosis to the date of death from any cause or last follow-up. PFS was defined as the time from diagnosis to disease progression (such as localized or metastatic disease recurrence or progression),death from any cause or last follow-up. Survival outcomes were estimated by Kaplan–Meier analysis, and group comparisons were performed using the log-rank test. Statistical analyses were carried out using R V4.1.3. (R Development Core Team, Vienna, Austria; http://www.rproject.org). Given the exploratory nature of the study, no adjustments for multiple testing were applied [27].

## Results

### Patient characteristics of the overall cohort

In this retrospective, multicenter analysis, we included 55 patients, who had been histopathologically and radiomorphologically diagnosed with resectable dMMR/MSI-H GEA. At data cut-off (December 14, 2025), median follow-up (time from initial cancer diagnosis to cut-off date) was 29.1 months (95% CI 21.1–40.4 months). Baseline tumor and patient characteristics are listed in Table 1. Clinical staging prior to therapeutic intervention had not been performed in one patient, as emergency surgery was performed due to tumor bleeding. In this case, pathological staging was used. All patients were dMMR and/or MSI-H. Testing was performed in 98% of patients (n = 54/55) via IHC (dMMR) and in 66% of patients (n = 36/55) via PCR (MSI-H). Two patients (4%) showed HER2 positivity (IHC 3 +).

**Table 1:** Patient characteristics of the overall cohort. CPS, combined positive score; FISH, fluorescence in-situ hybridization; GEJ, gastroesophageal junction; PCR, polymerase chain reaction; PD-L1, programmed-death ligand 1

Characteristic	N = 55^1^
*Sex*	
Female	22 (40%)
Male	33 (60%)
*Age*	
Mean (SD)	74 (10)
Median (Q1, Q3)	75 (68, 83)
Min, Max	47, 92
*Primary tumor site*	
esophagus	1 (2%)
GEJ	16 (29%)
stomach	38 (69%)
*Stage*	
1	11 (20%)
2	16 (29%)
2–3	6 (11%)
3	22 (40%)
*History of second malignancies*	
no	39 (71%)
yes	16 (29%)
*Mismatch repair status (IHC)*	
deficient	54 (100%)
not available	1
*Protein loss in IHC*	
MLH1, PMS2	41 (85%)
PMS2	3 (6%)
MLH1, PMS2, MLH6	2 (4%)
MLH1, PMS2, MSH2, MSH6	1 (2%)
MSH6, PMS2	1 (2%)
Not available	7
*Microsatellite analysis (PCR)*	
MSI-H	36 (100%)
not available	19
*HER2 status*	
0+	36 (67%)
1+	8 (15%)
2+ (FISH-)	8 (15%)
3+	2 (4%)
Unknown	1
*Signet ring cells*	
negative	49 (89%)
positive	6 (11%)
*PD-L1 CPS*	
< 1	2 (5%)
≥ 1	38 (95%)
Unknown	15
*PD-L1 CPS*	
< 5	5 (12%)
≥ 5	35 (88%)
Unknown	15
*PD-L1 CPS*	
< 10	12 (30%)
≥ 10	28 (70%)
Unknown	15

^1^n(%)

### Patient and tumor characteristics according to treatment subgroup

**Fig. 1:**
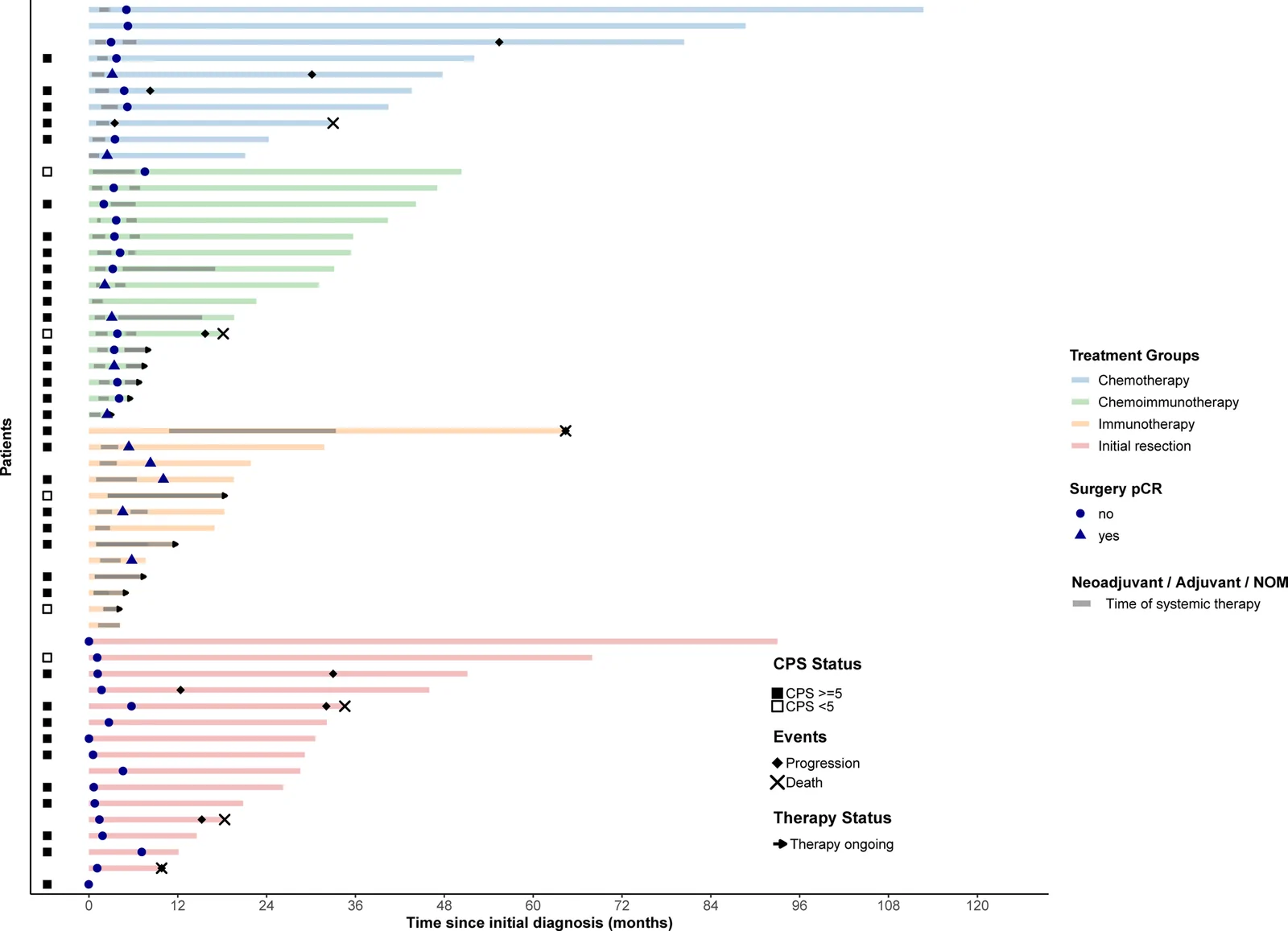
Swimmer plot showing treatment allocation and development since time of initial diagnosis for each patient. NOM, non-operative management; pCR, pathological complete response; CPS, combined positive score

Patient and tumor characteristics of the chemotherapy, the chemoimmunotherapy, immunotherapy, and the initial resection subgroup are summarized within Supplementary Table 1. Significant differences between the four groups were observed regarding age (*p* < 0.001), primary tumor site (*p* < 0.001), clinical stage (*p* = 0.001) and follow-up time (*p* = 0.003). Figure 1 visualizes the individual treatment and clinical outcome of the overall cohort according to treatment subgroup.

### Systemic therapies

Regarding preoperative treatment, the most common observed treatment strategy was FLOT (5-fluorouracil (5-FU)/leucovorin/oxaliplatin/docetaxel) in the chemotherapy group (60%, n = 6). In the chemoimmunotherapy group, all patients received anti-PD1/anti-PD-L1 inhibition combined with 5-FU/leucovorin/oxaliplatin ± docetaxel (FOLFOX or FLOT). One patient followed NOM-treatment with FLOT + atezolizumab.

All patients (n = 5/5) in the resected immunotherapy subgroup received neoadjuvant doublet ICI-inhibition, i.e. nivolumab + ipilimumab. One of those continued nivolumab + ipilimumab in an adjuvant setting. NOM in the immunotherapy subgroup consisted equally of anti–PD-1/anti–CTLA-4 combination therapy and anti-PD-1 monotherapy (50% each). Detailed treatment regimens and treatment durations are available in Table 2. No patient received adjuvant therapy without prior neoadjuvant systemic therapy.

**Table 2:** Systemic treatment regimens and treatment durations (median (IQR)) according to treatment subgroup (Chemotherapy only and Immunotherapy ± Chemo)

Characteristic	Neoadjuvant	Adjuvant	NOM
Chemotherapy only (n = 10)	9 (90%)	2 (20%)	1 (10%)
FLOT	6 (60%)		1 (100%)
FOLFOX	2 (20%)	2 (100%)	
Oxaliplatin/5-FU			
Oxaliplatin/Capecitabine			
Unknown	1 (10%)		
Treatment duration (months)	1.70 (1.40, 1.77)	1.0 (0.0, 1.9)	
Chemoimmunotherapy (n = 16)	15 (94%)	14 (88%)	1 (6%)
FLOT + Atezolizumab	5 (33)	3 (21%)	1 (100%)
FOLFOX + Atezolizumab	3 (20%)	4 (29%)	
FOLFOX + Nivolumab	2 (13%)		
FLOT + Durvalumab	2 (13%)	3 (21%)	
FLOT + Pembrolizumab	2 (13%)	3 (21%)	
FLOT + Nivolumab	1 (7%)	1 (7%)	
Treatment duration (months)	1.43 (1.40, 1.63)	1.4 (1.4, 2.8)	1.5 (1.5, 1.5)
Immunotherapy only (n = 13)	5 (38%)	1 (14%)	8 (62%)
Nivolumab + Ipilimumab	5 (100%)	1 (100%)	2 (25%)
Pembrolizumab			3 (38%)
Tremelimumab + Durvalumab			2 (25%)
Nivolumab			1 (12%)
Treatment duration (months)	2.33 (2.33, 2.80)	2.3 (2.3, 2.3)	4.6 (2.1, 11.7)

^1^n (%); Median (Q1, Q3)

FLOT, 5-fluorouracil/leucovorin/oxaliplatin/docetaxel; FOLFOX, 5-fluorouracil/leucovorin/oxaliplatin; 5-FU, 5-fluorouracil; NOM, non-operative management

### Treatment groups according to diagnosis by year

Cases of dMMR/MSI-H resectable GEA were identified between 2014 and 2025. 2022 marked the first year of diagnosis, in which most patients were treated with an immunotherapy-based regimen (n = 7/12, 58%). Patients diagnosed in 2025 received only immunotherapy-based treatment (n = 9/9, 100%). A non-statistically significant tendency towards the application of immunotherapy-based regimens within recent years was observed (p = 0.066). Treatment groups according to diagnosis by year are available within Supplementary Table 1.

### Surgery and NOM

Resection was performed in 45 patients (82%). In two patients of the initial resection subgroup, endoscopic mucosal resection was performed (n = 2/16).

After completing neoadjuvant treatment, nine out of ten patients (90%) of the chemotherapy subgroup underwent surgery. In one patient, resection was not completed due to intraoperatively detected peritoneal carcinomatosis, thus following NOM. Two patients (22%) achieved pCR.

In the chemoimmunotherapy group, resection was performed in 15/16 patients (94%). pCR rate was 27% (n = 4/15). In the immunotherapy group, surgery was performed in 5/13 patients (38%). Five out of 5 resected patients (100%) achieved pCR, all of whom received double checkpoint inhibition.

**Fig. 2:**
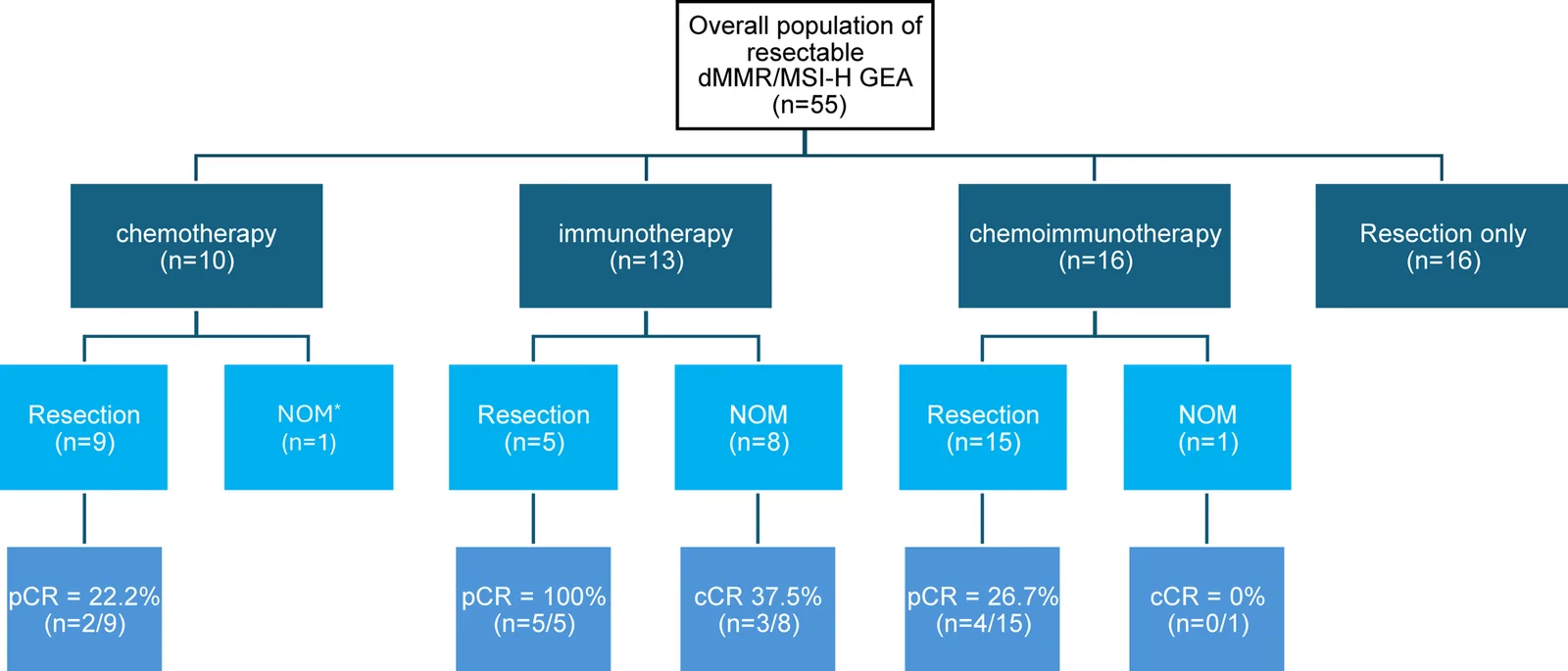
Patient allocation, treatment strategies and subsequent response rates. cCR, clinical complete response; dMMR, deficient mismatch repair; GEA, gastroesophageal adenocarcinoma; MSI-H, microsatellite instability-high; NOM, non-operative management; pCR, pathological complete response; * Non-operative management due to intraoperatively detected progression after neoadjuvant chemotherapy (peritoneal carcinomatosis)

Reasons for surgery omission and thus proceeding with NOM in 10 patients were various, independent of initial treatment intent: Patient refusal (n = 4, 40%), cCR and previous immune-related adverse events (irAE, n = 2, 20%), cCR and advanced age (n = 1, 10%), partial remission, advanced age, and non-eligibility for oncological surgery (n = 1, 10%), missing data (n = 1, 10%) and the above mentioned, intraoperatively observed progression (n = 1, 10%). Three patients with NOM in the immunotherapy group achieved cCR (38%, n = 3/8). No cCR was observed in the one patient receiving non-operative chemoimmunotherapy. Two patients treated with nivolumab + ipilimumab achieved cCR with no radiomorphogically measurable tumor on CT scan and had tumor-free rebiopsy. The third patient received initial neoadjuvant treatment with tremelimumab + durvalumab, but after cCR in the first restaging and no malignant cells within rebiopsy, resection was omitted. Primary tumor site and follow-up duration differed significantly between patients undergoing NOM and the remaining cohort: 9 of 10 patients in the NOM group had gastric primary tumor, and median follow-up was shorter in the NOM group (14.5 vs. 30.6 months; *p* = 0.012 and *p* = 0.031, respectively). Further baseline information regarding patients with NOM are available within Supplementary Table 1. Patient allocation, treatment strategies and subsequent response rates are summarized in Fig. 2.

A significant association between complete response (i.e. pCR and cCR combined) and a CPS ≥ 10 was observed (*p* = 0.027). Subgroup analyses for complete response according to clinical, histological, treatment and laboratory characteristics are shown in Supplementary Figure 1.

### PFS and OS according to treatment subgroup

**Fig. 3:**
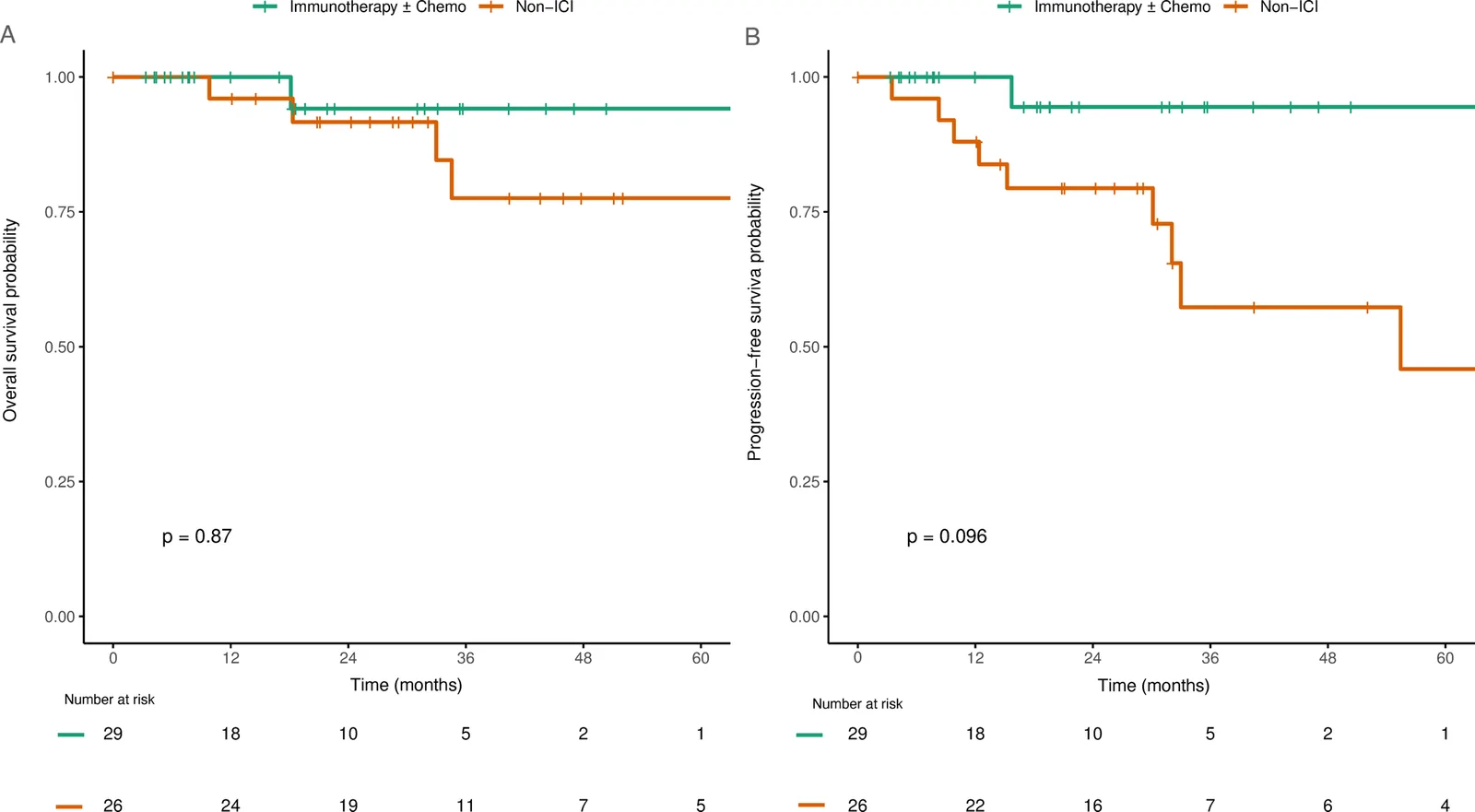
Kaplan Meier curves for overall (**A**) and progression-free (**B**) survival according to immune-checkpoint-inhibition based strategies, i.e. immunotherapy and chemoimmunotherapy combined, and non-immune-checkpoint-inhibition based strategies, i.e. chemotherapy and initial resection combined. ICI, immune-checkpoint inhibition

Median OS for patients with ICI-based regimens, i.e. immunotherapy ± chemotherapy, was 64.4 months (95% CI NA-NA months), while in the other group, i.e. combined chemotherapy only and initial resection, median OS was not reached (95% CI NA-NA months) which is shown within Fig. 3A (*p* = 0.87). Median PFS for patients treated with ICI-based regimens was 64.4 months (95% CI NA-NA months), while in all patients with non-ICI based treatment, median PFS was 55.4 months (95% CI 32.1-NA months, *p* = 0.096, Fig. 3B). Median follow-up for immunotherapy-based regimens was 19.6 months (95% CI 12.0–33.1 months) and for non-ICI based treatment 40.4 months (95% CI 29.1–68.0 months).

In the ICI-based treatment group, median OS and PFS were reached at 64.4 months after initial cancer diagnosis, corresponding to the event time of the last patient at risk, after which no patients remained under observation.

36 months OS- and PFS-rates were 88% and 67% for the chemotherapy only group, 91% and 91% in the chemoimmunotherapy group, 100% and 100% in the immunotherapy group and 68% and 42% in the initial resection group, respectively.

Kaplan–Meier curves for the OS and PFS according to treatment group are visualized in Supplementary Fig. 2.

## Discussion

This exploratory, multicenter, real-world analysis showed that the management of resectable dMMR/MSI-H GEA in Austria has shifted significantly over time, with immunotherapy-based strategies increasingly adopted in both perioperative and definitive settings. Pre- and non-operative immunotherapy with or without a chemotherapy-backbone was observed to show a strong trend toward meaningful antitumoral activity within this retrospective study. Our multicenter patient population constitutes a highly representative and, to the best of our knowledge, the largest reported real-world cohort with characteristics comparable to those described in international cohorts, such as high median age, low HER2-positivity occurrence, and high CPS values [9, 28]. We emphasize that this analysis is the first to evaluate multiple treatment approaches within a single real-world cohort, which has not yet been investigated in other real-world cohorts and has so far been evaluated only in small phase II trials. [23, 24].

Some phase II trials observed substantial antitumor activity of immunotherapy-based therapy regimens in resectable dMMR/MSI-H GEA, however, presumably due to the low frequency of this molecular subtype, no phase III trials comparing different treatment strategies in this setting have been conducted yet [14, 15, 29]. Thus, consensus on European-level guideline is still lacking [22]. Our analysis may support decision-making in daily clinical routine.

Neoadjuvant doublet-ICI inhibition showed promising antitumor activity in our cohort, as all patients achieved pCR (5/5), exceeding the near 60% pCR-rate in the NEONIPIGA-trial (n = 17/29) and the INFINITY-trial (n = 9/15), respectively [14, 15]. In the case of non-operative management, cCR was only observed in patients with doublet-ICI inhibition (n = 3/4), whereas an Italian real-world cohort reported a 100% cCR with anti-PD1-therapy alone (n = 4/4) [23]. Although no direct comparison between ICI monotherapy and doublet therapy exists in this patient population, anti-PD1 monotherapy remains controversial, as neoadjuvant short course (1–2 cycles) pembrolizumab fell short of expectations concerning pCR rate in the phase-II trial for dMMR/MSI-H GEA [30]. Therefore, not only the treatment composition (ICI monotherapy versus ICI doublet) but also treatment duration appears to be relevant for achieving a cCR/pCR.

Patients undergoing preoperative chemoimmunotherapy in our real-world cohort had a pCR-rate of 27% (n = 4/15), which is comparable to the findings reported for MSI-H patients in the phase III MATTERHORN trial (28%, n = 7/25) [18]. Yet, the pCR-rate of chemoimmunotherapy considerably varied across trials, with other phase-II trials reporting rates of 17–80% [29, 31, 32]. Consequently, the need of a chemotherapy-backbone remains a matter of debate. To date, no head-to-head comparison between single- or doublet-immunotherapy versus chemoimmunotherapy has been made in randomized trials, which warrants further investigation.

ICI-based treatment regimens were associated with a numerically longer progression-free survival. One possible confounder explaining the lack of superiority is the earlier clinical stage of patients undergoing initial resection, which is generally associated with improved outcomes and may have led to baseline imbalances between the groups. Furthermore, these estimates remain immature, similarly to data from other trials [14, 15, 29], and since the follow-up time significantly varied between each treatment subgroup, particularly between ICI- and non-ICI-based regimens in our cohort, these data should therefore be interpreted with caution. Nevertheless, these findings altogether suggest the feasibility of ICI-based strategies in this selected patient population [33].

Older patients were more likely to receive pre- or non-operative immunotherapy, or to undergo initial surgical resection, aiming at avoiding the side effects of chemotherapy-based regimens. Tumors located in the stomach as well as tumors with earlier clinical stage were more likely to undergo initial resection.

In patients with chemotherapy followed by resection, pCR was observed in two of 9 cases (22%), which suggests that a fluoropyrimidine/platinum-based doublet with or without taxane shows activity in some patients with dMMR/MSI-H GEA, similarly to other trials (8–27%) [18, 31]. Yet the specific subset of patients who derive benefit from chemotherapy alone remains to be defined, as long-term toxicity after triplet combination chemotherapy poses a major concern [18, 31]. However, careful attention to irAE is also warranted and toxicity in patients receiving immunotherapy should not be underestimated. In our cohort, two patients did not undergo surgery due to higher grade side effects of doublet ICI-inhibition, leading to one discontinuation of treatment. Toxicities also reinforce the discussion of therapy duration, as prolonged exposure increases the risk of developing irAE [9]. Particularly in the case of organ-preserving, non-operative management, further data are necessary to mark an endpoint of ICI-inhibition [23]. Yet, reduced surgical morbidity, prevention of treatment-related sarcopenia, and preservation of functional capacity in everyday life represent key arguments in favor of a non-operative approach [34, 35].

Therefore, decision-making in daily clinical routine may become increasingly patient-specific, depending not only on individual patient and tumor characteristics, but on patient demand, too. In our analysis, a statistically significantly higher complete response rate (cCR/pCR) was documented among tumors with a PD-L1 CPS ≥ 10 compared to those with a CPS < 10 (45% versus 0%, Supplementary Figure 1). In contrast to our findings, Fazio et al. described a numerically higher complete response rate among patients with a PD-L1 CPS < 10 in their real-world cohort [23]. Consequently, expression of PD-L1 may not be the ideal biomarker for stratifying immunotherapy sensitivity in dMMR/MSI-H tumors, as even within this subgroup, there seems to be heterogeneity regarding higher responsiveness to immunotherapy [36]. Kwon et al. demonstrated that abundance of infiltrating T-cells, greater T-cell receptor clonal diversity, and fewer stem-like exhausted T cells at initial diagnosis seem to be potent predictive features for response to anti-PD1 inhibitors, whereas PD-L1 CPS alone did not reliably distinguish responders from non-responders in this cohort of MSI-H gastric cancer [36]. Increased PD-1 + CD8 + T-cells, which correlated with durable clinical benefit, may represent another non-invasive, peripheral blood-based and consequently easily available biomarker [36]. Therefore, future translational strategies should prospectively investigate multidimensional biomarker assessment including tumor-intrinsic features and peripheral immune parameters. Such approaches may contribute to the identification of patients with dMMR/MSI-H GEA who may achieve durable benefit from ICI-monotherapy, while also identifying subgroups that may require escalated or combination therapy approaches.

Due to the retrospective nature of this analysis, detailed adverse event data and quality of life cannot be obtained. Furthermore, the relatively small sample size limits statistical power and thus the generalizability of our findings. Nonetheless, this exploratory study represents to the best of our knowledge the largest reported and highly representative resectable dMMR/MSI-H GEA cohort with diverse treatment strategies in a real-world setting. Meaningful antitumor activity of neoadjuvant or non-operative immunotherapy-based regimens, particularly of doublet ICI-inhibition, was observed in patients with resectable dMMR/MSI-H GEA. Yet, several questions remain to be elucidated, since guideline-based treatment recommendations for this subgroup are lacking: Which exact regimen should case-managing physicians pursue, when confronted with such a patient in daily clinical routine? Is a chemotherapy-backbone necessary? Is single-agent immunotherapy as effective as doublet immunotherapy? When is it reasonable to omit surgery and pursue an organ-preserving management? What is the optimal treatment duration, particularly in non-operatively managed patients? Thus, future clinical trials and additional real-world data are indispensable to provide treatment recommendations in resectable dMMR/MSI-H GEA.

## Supplementary Material

Below is the link to the electronic supplementary material.Supplementary File 1 (DOCX 604 kb)

## Data Availability

Further data will be shared upon reasonable request to the corresponding author.

## Abbreviations

cCR: Clinical complete response
CPS: Combined Positive Score
dMMR: Deficient mismatch repair
ESMO: European Society For Medical Oncology
GEA: Gastroesophageal adenocarcinoma
MSI-H: Microsatellite instability-high
NGS: Next-generation sequencing
OS: Overall survival
ICI: Immune checkpoint inhibition
IHC: Immunohistochemistry
irAE: Immune-related adverse events
pCR: Pathological complete response
PCR: Polymerase chain reaction
PD-(L)1: Programmed-death (ligand) 1
PFS: Progression-free survival

## Declarations

### Statement on the use of artificial intelligence

Artificial intelligence (AI)-based tools were used to support specific components of this study. ChatGPT (OpenAI, GPT-5) was utilized to assist with R programming for statistical analyses, data visualization, and figure generation. All aspects of conceptualization, study design, data interpretation, and critical reasoning were performed by the authors. All AI-assisted outputs were thoroughly reviewed, verified, and edited by the authors, who take full responsibility for the accuracy and integrity of the final manuscript.
