# Supplementary material for: Therapeutic management of resectable mismatch-repair deficient/microsatellite instability-high gastroesophageal adenocarcinoma: a real-world analysis from Austria
Source: Gastric Cancer. 2026 Jul 23;29(5):949–59. doi: 10.1007/s10120-026-01776-1 (PMC13525024; doi:10.1007/s10120-026-01776-1)
Supplement: Supplementary file 1 [file 10120_2026_1776_MOESM1_ESM.docx]

**Supplementary Table 1. Patient characteristics according to treatment subgroup.** CPS, combined positive score; FISH, fluorescence in situ hybridization; GEJ, gastroesophageal junction; IHC, immunohistochemistry; MSI-H, microsatellite instability-high, PCR, polymerase chain reaction

| **Characteristic** | **Chemotherapy**   N = 10^1^ | **Chemoimmunotherapy**  N = 16^1^ | **Immunotherapy**   N = 13^1^ | **Initial resection**  N = 16^1^ | **p-value**^2^ |
| --- | --- | --- | --- | --- | --- |
| **Sex** |  |  |  |  | 0.70 |
| Female | 3 (30%) | 6 (38%) | 7 (54%) | 6 (38%) |  |
| Male | 7 (70%) | 10 (63%) | 6 (46%) | 10 (63%) |  |
| **Age** |  |  |  |  | <0.001 |
| Mean (SD) | 72 (8) | 64 (9) | 80 (8) | 81 (6) |  |
| Median (Q1, Q3) | 72 (67, 77) | 65 (57, 71) | 82 (75, 85) | 81 (78, 86) |  |
| Min, Max | 61, 85 | 47, 83 | 68, 90 | 68, 92 |  |
| **Primary tumor site** |  |  |  |  | <0.001 |
| esophagus | 0 (0%) | 1 (6%) | 0 (0%) | 0 (0%) |  |
| GEJ | 6 (60%) | 8 (50%) | 2 (15%) | 0 (0%) |  |
| stomach | 4 (40%) | 7 (44%) | 11 (85%) | 16 (100%) |  |
| **Stage** |  |  |  |  | 0.001 |
| 1 | 0 (0%) | 0 (0%) | 2 (15%) | 9 (56%) |  |
| 2 | 2 (20%) | 6 (38%) | 3 (23%) | 5 (31%) |  |
| 2-3 | 3 (30%) | 2 (13%) | 1 (8%) | 0 (0%) |  |
| 3 | 5 (50%) | 8 (50%) | 7 (54%) | 2 (13%) |  |
| **History of second malignancies** |  |  |  |  | 0.40 |
| no | 9 (90%) | 12 (75%) | 8 (62%) | 10 (63%) |  |
| yes | 1 (10%) | 4 (25%) | 5 (38%) | 6 (38%) |  |
| **Mismatch repair status (IHC)** |  |  |  |  | >0.99 |
| deficient | 10 (100%) | 16 (100%) | 12 (100%) | 16 (100%) |  |
| unknown | 0 | 0 | 1 | 0 |  |
| **Protein loss in IHC** |  |  |  |  | 0.78 |
| MLH1, PMS2 | 8 (100%) | 13 (87%) | 9 (90%) | 11 (73%) |  |
| MLH1, PMS2, MSH6 | 0 (0%) | 1 (7%) | 0 (0%) | 1 (7%) |  |
| MLH1, PMS2, MSH2, MSH6 | 0 (0%) | 0 (0%) | 0 (0%) | 1 (7%) |  |
| MSH6, PMS2 | 0 (0%) | 0 (0%) | 1 (10%) | 0 (0%) |  |
| PMS2 | 0 (0%) | 1 (7%) | 0 (0%) | 2 (13%) |  |
| not available | 2 | 1 | 3 | 1 |  |
| **Microsatellite analysis (PCR)** |  |  |  |  | 0.26 |
| MSI-H | 7 (100%) | 10 (100%) | 6 (100%) | 13 (100%) |  |
| not available | 3 | 6 | 7 | 3 |  |
| **Signet ring cells** |  |  |  |  | 0.75 |
| negative | 8 (80%) | 14 (88%) | 12 (92%) | 15 (94%) |  |
| positive | 2 (20%) | 2 (13%) | 1 (8%) | 1 (6%) |  |
| **CPS cut-off 1** |  |  |  |  | 0.99 |
| <1 | 0 (0%) | 1 (7%) | 0 (0%) | 1 (9%) |  |
| ≥1 | 5 (100%) | 13 (93%) | 10 (100%) | 10 (91%) |  |
| Unknown | 5 | 2 | 3 | 5 |  |
| **CPS cut-off 5** |  |  |  |  | 0.84 |
| <5 | 0 (0%) | 2 (14%) | 2 (20%) | 1 (9%) |  |
| ≥5 | 5 (100%) | 12 (86%) | 8 (80%) | 10 (91%) |  |
| Unknown | 5 | 2 | 3 | 5 |  |
| **CPS cut-off 10** |  |  |  |  | 0.59 |
| <10 | 1 (20%) | 5 (36%) | 3 (30%) | 3 (27%) |  |
| ≥10 | 4 (80%) | 9 (64%) | 7 (70%) | 8 (73%) |  |
| Unknown | 5 | 2 | 3 | 5 |  |
| **Follow-up in months** |  |  |  |  | 0.003 |
| Mean (SD) | 54 (30) | 26 (16) | 18 (16) | 32 (23) |  |
| Median (Q1, Q3) | 46 (33, 80) | 27 (8, 38) | 17 (8, 20) | 29 (16, 40) |  |
| Min, Max | 21, 113 | 3, 50 | 4, 64 | 0, 93 |  |
| ^1^n (%) | | | | | |
| ^2^Fisher's exact test; Kruskal-Wallis rank sum test | | | | | |

**Supplementary Table 2. Treatment subgroups according to year of initial cancer diagnosis**

| **Characteristic** | **Chemotherapy** N = 10^1^ | **Chemoimmunotherapy** N = 16^1^ | **Immunotherapy**  N = 13^1^ | **Initial resection**  N = 16^1^ | **p-value**^2^ |
| --- | --- | --- | --- | --- | --- |
| **Year of initial cancer diagnosis** |  |  |  |  | 0.066 |
| 2014 | 1 (10%) | 0 (0%) | 0 (0%) | 0 (0%) |  |
| 2016 | 0 (0%) | 0 (0%) | 0 (0%) | 1 (6.3%) |  |
| 2017 | 1 (10%) | 0 (0%) | 0 (0%) | 0 (0%) |  |
| 2018 | 0 (0%) | 0 (0%) | 0 (0%) | 2 (13%) |  |
| 2019 | 1 (10%) | 0 (0%) | 1 (7.7%) | 2 (13%) |  |
| 2020 | 1 (10%) | 0 (0%) | 0 (0%) | 0 (0%) |  |
| 2021 | 2 (20%) | 1 (6.3%) | 0 (0%) | 4 (25%) |  |
| 2022 | 2 (20%) | 6 (38%) | 1 (7.7%) | 3 (19%) |  |
| 2023 | 1 (10%) | 3 (19%) | 3 (23%) | 2 (13%) |  |
| 2024 | 1 (10%) | 1 (6.3%) | 4 (31%) | 2 (13%) |  |
| 2025 | 0 (0%) | 5 (31%) | 4 (31%) | 0 (0%) |  |
| ^1^n (%) | | | | | |
| ^2^Pearson's Chi-squared test | | | | | |

**Supplementary Table 3. Baseline patient and tumor characteristics for Non-Operative Management (NOM) patients compared to the remaining cohort.**
CPS, combined positive score; FISH, fluorescence in situ hybridization; GEJ, gastroesophageal junction; HER2, human epidermal growth factor receptor 2; MSI-H, microsatellite instability-high; IHC, immunohistochemistry, PCR, polymerase chain reaction

| **Characteristic** | **NOM**  N = 10^1^ | **Remaining cohort**  N = 45^1^ | **p-value**^2^ |
| --- | --- | --- | --- |
| **Sex** |  |  | 0.070 |
| Female | 7 (70%) | 15 (33%) |  |
| Male | 3 (30%) | 30 (67%) |  |
| **Age** |  |  | 0.12 |
| Mean (SD) | 79.2 (9.5) | 73.2 (10.3) |  |
| Median (Q1, Q3) | 81.0 (70.0, 88.0) | 74.0 (68.0, 82.0) |  |
| Min, Max | 65.0, 90.0 | 47.0, 92.0 |  |
| **Age categories** |  |  | 0.75 |
| <60 | 0 (0%) | 5 (11%) |  |
| ≥70 | 8 (80%) | 29 (64%) |  |
| 60–69 | 2 (20%) | 11 (24%) |  |
| **Primary tumor site** |  |  | **0.012** |
| esophagus | 1 (10%) | 0 (0%) |  |
| GEJ | 0 (0%) | 16 (36%) |  |
| stomach | 9 (90%) | 29 (64%) |  |
| **Stage** |  |  | 0.74 |
| 1 | 2 (20%) | 9 (20%) |  |
| 2 | 4 (40%) | 12 (27%) |  |
| 2-3 | 0 (0%) | 6 (13%) |  |
| 3 | 4 (40%) | 18 (40%) |  |
| **History of second malignancies** |  |  | 0.71 |
| no | 8 (80%) | 31 (69%) |  |
| yes | 2 (20%) | 14 (31%) |  |
| **Mismatch repair status (IHC)** |  |  | 0.18 |
| deficient | 9 (90%) | 45 (100%) |  |
| unknown | 1 (10%) | 0 (0%) |  |
| **Protein loss in IHC** |  |  | 0.11 |
| MLH1, PMS2 | 5 (71%) | 36 (88%) |  |
| MLH1, PMS2, MSH6 | 1 (14%) | 1 (2%) |  |
| MLH1, PMS2, MSH2, MSH6 | 0 (0%) | 1 (2%) |  |
| MSH6, PMS2 | 1 (14%) | 0 (0%) |  |
| PMS2 | 0 (0%) | 3 (7%) |  |
| Not available | 3 | 4 |  |
| **Microsatellite analysis (PCR)** |  |  |  |
| MSI-H | 7 (100%) | 29 (100%) |  |
| Not available | 3 | 16 |  |
| **HER2 status** |  |  | 0.92 |
| 0 | 7 (70%) | 29 (66%) |  |
| 1+ | 2 (20%) | 6 (14%) |  |
| 2+ (FISH-) | 1 (10%) | 7 (16%) |  |
| 3+ | 0 (0%) | 2 (4%) |  |
| Not available | 0 | 1 |  |
| **Signet ring cells** |  |  | 0.58 |
| negative | 10 (100%) | 39 (87%) |  |
| positive | 0 (0%) | 6 (13%) |  |
| **CPS cut-off 5** |  |  | 0.31 |
| <5 | 2 (22%) | 3 (10%) |  |
| ≥5 | 7 (78%) | 28 (90%) |  |
| Not available | 1 | 14 |  |
| **CPS cut-off 10** |  |  | >0.99 |
| <10 | 3 (33%) | 9 (29%) |  |
| ≥10 | 6 (67%) | 22 (71%) |  |
| Not available | 1 | 14 |  |
| **Disease progression** |  |  | >0.99 |
| no | 8 (80%) | 36 (80%) |  |
| yes | 2 (20%) | 9 (20%) |  |
| **Treatment duration (months)** |  |  | 0.33 |
| Mean (SD) | 7.0 (7.6) | 3.6 (3.1) |  |
| Median (Q1, Q3) | 3.0 (2.0, 7.1) | 2.8 (1.9, 3.8) |  |
| Min, Max | 1.5, 22.8 | 1.4, 14.1 |  |
| Not available | 1 | 17 |  |
| **Follow-up (months)** |  |  | **0.031** |
| Mean (SD) | 18.9 (18.5) | 33.6 (24.6) |  |
| Median (Q1, Q3) | 14.5 (5.3, 22.6) | 30.6 (18.3, 44.2) |  |
| Min, Max | 4.2, 64.4 | 0.0, 112.7 |  |
| ^1^n (%) | | | |
| ^2^Fisher's exact test; Wilcoxon rank sum test | | | |

**Supplementary Figure 1. Forest Plot of complete remission (pathological and clinical complete remission) by subgroup for clinical and histological characteristics (A) and treatment and laboratory characteristics (B).** CPS, combined positive score; CR, complete response; CRP, C-reactive protein (median 0.60 mg/dl); CT, chemotherapy; GEJ, gastroesophageal junction; IO, immunotherapy; NLR, neutrophil-lymphocyte ratio (median 3.31 G/L); PD-L1, programmed-death ligand 1; PLR, platelet-lymphocyte ratio (median 194.41 G/L)

**Supplementary Figure 2. Kaplan-Meier curves for overall (A) and progression-free survival (B) according to treatment group.**
